# Chronotype, sleep quality, depression and pre‐sleep rumination: A diary and actigraphy study

**DOI:** 10.1111/ejn.16551

**Published:** 2024-10-05

**Authors:** Efthymia Lamprou, Liia M. M. Kivelä, Jos H. T. Rohling, Johanna H. Meijer, Willem van der Does, Niki Antypa

**Affiliations:** ^1^ Department of Clinical Psychology, Institute of Psychology Leiden University Leiden The Netherlands; ^2^ Laboratory for Neurophysiology, Department of Cell and Chemical Biology Leiden University Medical Center Leiden The Netherlands; ^3^ Department of Psychiatry Leiden University Medical Center Leiden The Netherlands; ^4^ Leiden Institute of Brain and Cognition Leiden University Leiden The Netherlands; ^5^ Department of Psychology Panteion University of Social and Political Sciences Athens Greece

**Keywords:** depression, eveningness, morningness, rumination, sleep quality

## Abstract

Eveningness has been associated with both disturbed sleep and depression. It is unclear, however, if deprived sleep explains evening types' vulnerability to depression. The role of pre‐sleep rumination in these associations also remains understudied. The present study assessed the relationship between eveningness and sleep quality, as well as the possible mediating effect of pre‐sleep rumination and the moderating effect of a history of depression, under naturalistic conditions. Eighty‐eight Dutch‐speaking participants (87.5% females, 21.4 ± 3.7 years) were selected on the basis of their non‐intermediate chronotype using the Morningness Eveningness Questionnaire (evening types (*n* = 53); morning types (*n* = 35)). Depression status was assessed through a diagnostic interview (healthy (*n* = 61); remitted depressed (*n* = 27)). Participants' sleep characteristics were monitored via actigraphy and sleep diaries for seven consecutive days and nights. Pre‐sleep rumination was measured via a self‐report questionnaire. Evening types had longer subjective and actigraphic sleep onset latency than morning types. Pre‐sleep rumination did not mediate the former associations but predicted longer subjective sleep onset latency. Furthermore, the relationship between chronotype and subjective sleep onset latency was moderated by depression history. Remitted depressed evening types reported longer sleep onset latency than healthy evening and morning types, possibly posing the former at a higher risk for depressive relapse. Overall, the current findings address the need to further investigate the physiological signature of circadian rhythms and sleep latency. This could serve as a foundation for the development of prevention and early intervention programs, tailored for mood and sleep disorders.

## Graphical abstract

Evening types have a longer sleep onset latency that is not related to pre‐sleep rumination. The longest sleep onset delay is observed in evening types with a depression history, posing this group at higher risk for depressive relapse.

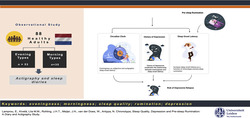

**Abbreviations**
SOL: Sleep Onset Latency
TST: Total Sleep Duration
WASO: Wakefulness After Sleep Onset
M‐types: Morning types
I‐types: Intermediate types
E‐types: Evening types
PSG: Polysomnography
MEQ: Morningness Eveningness Questionnaire
RSS: Rumination Response Scale
M.I.N.I.: Mini International Neuropsychiatric Interview
DFA: Detrended Fluctuation Analysis
MDD: Major Depressive Disorder
(M)ANOVA: (Multivariate) Analysis of Variance

## 1 INTRODUCTION

Chronotype refers to individual differences in circadian rhythmicity and can be classified into three types: the morning (M‐type), evening (E‐type) and intermediate type (I‐type; Horne & Ostberg, 1976). Given the crucial role that circadian rhythms play in facilitating sleep, chronotype has been linked to sleep characteristics (Poon et al., 2024; Soehner et al., 2011). E‐types have more insomnia symptoms and nightmare frequency and nightmare distress than M‐types and they also use more sleep medications (Choo et al., 2023; Merikanto et al., 2012). Moreover, they have poorer sleep quality, indexed by a longer self‐reported sleep onset latency (SOL), shorter total sleep time (TST) and more awakenings after sleep onset (WASO) (Roeser et al., 2012; Sun et al., 2019; Tzischinsky & Shochat, 2011). Overall, E‐types seem to be more vulnerable to sleep disturbances than M‐types.

Most studies have only used subjective measurements of sleep (Kitamura et al., 2010; Roeser et al., 2012; Tzischinsky & Shochat, 2011), however, participants may report sleep disturbances that are not confirmed by objective assessments (Pillai et al., 2014). Actigraphy‐based studies on the association between eveningness and sleep parameters have produced mixed results. Young adult E‐types have lower sleep efficiency than M‐types (Lehnkering & Siegmund, 2007) and shorter sleep duration during weekdays (e.g., Brooks et al., 2021). Furthermore, in a sample of older adults, eveningness was associated with longer sleep onset latency (SOL), less sleep efficacy and more awakenings after sleep onset (Sauers et al., 2023). However, no significant differences among chronotypes in actigraphic sleep onset latency (SOL), total sleep time (TST) and wakefulness after sleep onset (WASO) have also been observed (e.g., Martin et al., 2012; Natale et al., 2022; Rosa et al., 2021), although E‐types often report lower subjective sleep quality than M‐types (e.g., Martin et al., 2012). Along these lines, a recent actigraphic study by Tonetti et al. (2024), in children and adolescents, proposed that even though chronotypes significantly differ in terms of sleep quantity (i.e., TST) and timing(i.e., bedtime, get‐up time and midpoint of sleep), they are not distinct with regards to objective sleep quality (i.e., sleep efficiency, SOL, WASO). Hence, it seems that eveningness is frequently linked with self‐reported sleep problems, but findings are not robust with objective sleep measurements.

Eveningness and sleep disturbances have also been independently associated with depression. Specifically, eveningness has consistently been associated with a higher prevalence of depressive symptoms (e.g., Hidalgo et al., 2009; Kivelä et al., 2018; Levandovski et al., 2011; Merikanto et al., 2013), current or past diagnosis of major depression (MDD) and treatment with antidepressant medication (e.g., Antypa et al., 2016; Merikanto et al., 2015). Eveningness was also a risk factor for later depression in longitudinal studies (Haraden et al., 2017; Vetter et al., 2018). Similarly, recent evidence suggests that M‐types are less likely to exhibit depressive symptoms compared to their evening counterparts, and this relationship is mediated by better sleep quality in M‐types (Poon et al., 2024). Disturbed sleep is one of the diagnostic criteria of depression but may also be a precursor, as it is associated with an increased risk of depression onset (Baglioni et al., 2011), and depression relapse (Li et al., 2012).

It remains unclear if sleep disturbances, which are often more pronounced in E‐types (Roeser et al., 2012), explain E‐types' vulnerability to depression (e.g., Lang et al., 2022). A recent longitudinal study of 742 Dutch students found that poor sleep quality mediated the association between eveningness and depression, even after one year of follow‐up (Van den Berg et al., 2018). In contrast, others have shown that eveningness and insomnia are independent risk factors for non‐remission of depression (Chan et al., 2014). The paths through which disturbed sleep increases E‐types' susceptibility to depression remain unclear.

Recent evidence implicates rumination in the aetiology of disturbed sleep (Pillai et al., 2014). Rumination refers to the unproductive and repetitive focus on the self or negative affect states and may also play a role in the chronotype‐sleep association, as evening types are known to be more prone to rumination (Antypa et al., 2017). Individuals with a high propensity to ruminate (high‐trait ruminators) reported worse sleep quality than low‐trait ruminators following an experimentally‐induced stressful life event (Guastella & Moulds, 2007). In another study, ruminative thoughts predicted longer subjective and objective sleep onset latency (SOL) in the night following an acute psychosocial stressor, particularly for high‐trait ruminators (Zoccola et al., 2009). A limitation of both of these studies is that neither established whether rumination occurred *naturally* before sleep and was based on single‐night assessments. In contrast, a seven‐day actigraphy study in students with depressive symptomatology, pre‐sleep rumination predicted both longer subjective and actigraphic SOL (Pillai et al., 2014).

Interestingly, a recent online pilot study explored the role of rumination on sleep quality and depression across M‐types and E‐types (Nowakowska‐Domagała et al., 2022). The study found that having difficulty adapting to different times of the day, regardless of morningness‐eveningness preference and poor morning effect was linked to higher levels of rumination. However, eveningness by itself was not directly related to increased rumination (Nowakowska‐Domagała et al., 2022). The latter partially mediated the relationship between the morning effect and sleep quality, as well as depression and sleep quality, respectively. On the other hand, Tian et al. (2019) proposed that poor sleep quality in E‐types could be attributed to their higher inclination to ruminate, indexed by hyperarousal of the default mode network. This evidence holds the premise that rumination might be the key to unravelling the multifaceted relationship of poor sleep quality and eveningness (Bauducco et al., 2020). Yet the limited and partially conflicting findings warrant further exploration.

This is the first study to investigate both subjective and objective sleep parameters, along with pre‐sleep rumination, in morning and evening types. The study was conducted in young adults who were either healthy or had a history of depression. Using a seven‐day multi‐method approach (sleep diaries and actigraphy), we hypothesized that: (i) E‐types have longer SOL than M‐types; (ii) the effect on SOL is mediated by pre‐sleep rumination and (iii) moderated by history of depression. Finally, exploratory analyses were conducted between chronotype and sleep maintenance factors (WASO and TST) hypothesizing that (iv) E‐types will experience longer WASO and shorter TST, compared to M‐types. Alcohol and caffeine consumption were also assessed as potential cofounders of the above associations since they have been associated with chronotype (with increased use among E‐types; Taylor et al., 2011).

## 2 MATERIALS AND METHODS

The current study was an observational study, approved by Leiden University Psychology Ethics Committee on 26‐1‐2015; CEP number‐7247102255.

### 2.1 Participants

Ninety Dutch‐speaking participants were recruited via advertisements in Leiden University and the city of Leiden. Participants were selected based on their chronotype as assessed with the Morningness‐Eveningness Questionnaire (MEQ; Horne & Ostberg, 1976). Only E‐types (*n* = 55) and M‐types (*n* = 35) were included. All participants provided written informed consent. The inclusion criteria were: age between 18 and 45 years; fluency in Dutch; no (history of a) psychiatric disorder except for past depressive episode, according to DSM‐IV ([APA] American Psychiatric Association, 2000). Psychiatric diagnoses were assessed with MINI International Neuropsychiatric Interview (M.I.N.I.; Sheehan et al., 1998). Medication dosage, if any, had to be stable.

Two participants were excluded as they did not complete any of the three measurements (Sleep Diary, Actigraphy and Rumination Response Scale). Of the remaining 88 participants (E‐types = 53; M‐types = 35), two either had not filled the sleep diary at all or not according to the instructions, and one significantly deviated (>7 SDs) from the other participants regarding the diary‐based alcohol consumption. Seven did not wear the actigraph or had less than three days of recording, and two did not fill in the Rumination Response Scale. This yielded a final sample of 86 for subjective sleep and pre‐sleep rumination analyses, and 81 for actigraphy‐based analyses.

### 2.2 Materials

#### 2.2.1 General information

Data on age, gender, education, working status and family status were collected via a 20‐item questionnaire.

#### 2.2.2 Chronotype

Chronotype was assessed with the Morningness Eveningness Questionnaire (MEQ; Horne & Ostberg, 1976). The MEQ consists of 19 items, which are questions regarding preferred wake‐up and bedtimes and daily activity schedules (Horne & Ostberg, 1976). Scores <42 define E‐types, while scores >58 indicate M‐types. Chronotype was used as a dichotomous variable: E‐types (coded as 0) and M‐types (coded as 1).

#### 2.2.3 Psychiatric assessment

The expanded version of the Mini International Neuropsychiatric Interview (M.I.N.I.; Sheehan et al., 1998) is a comprehensive diagnostic semi‐structured interview that was used to assess current and lifetime psychiatric diagnoses. In the present study, history of depression was utilized as a dichotomous variable: participants' status was classified as “healthy” (coded as 0) and “past depression” (coded as 1).

#### 2.2.4 Sleep diaries

Participants were instructed to fill a 26‐item sleep diary every morning (Carney et al., 2012), in order to subjectively measure SOL (i.e., “How long did it take to fall asleep?”) in minutes, WASO (i.e., “How long did the periods of waking last in total?”) in minutes and TST (i.e., “How long have you slept in total?”) in hours. All three sleep parameters were averaged across seven days, and the averaged values were used for the analyses.

#### 2.2.5 Actigraphy

Participants were instructed to wear the actigraph on the wrist of the non‐dominant hand for a week. Actigraphs are small motion detectors (accelerometers) that distinguish the sleep–wake cycles based on algorithms. In the present study, the MotionWatch 8 (CamNTech, Cambridge, UK) was utilized, which weighed 9.1 g with a standard wrist strap and was programmed to sample motion per 5‐second epochs, as recommended for seven days recording with light recording enabled. Moreover, a standard threshold of motion detection was applied, ranging between 0.01 g and 8 g, along with 3–11 Hz bandwidth filtering. The participants were asked to press the event marker when they went to bed with the intention to sleep and again when they woke up. The MotionWare software was used to conduct the sleep–wake analyses (CamNTech, Cambridge, UK). SOL was the total time in minutes between the first event marker and the first epoch coded as sleep. TST was the total sleep time in hours between the first sleep epoch and the second event marker. Finally, WASO was the total time in minutes that was scored as wakefulness between the first sleep epoch and the second event marker. The event markers were cross‐referenced with the sleep diaries when one of the two event markers was not pressed. All three sleep parameters were averaged across the week, and the averaged values were used for the analyses.

Previous studies also investigated actigraphy data for self‐similarity in the data across different time scales as an indication of a healthy daily rhythm in humans (Goldberger et al., 2002; Hu et al., 2007; Hu et al., 2009; Joustra et al., 2018; Pittman‐Polletta et al., 2013). Correlations of activity fluctuations across different time scales can be assessed using a method called detrended fluctuation analysis (DFA). In short, DFA is used to calculate an average fluctuation value for different time windows, which is then plotted on a log–log scale. A straight line in this plot shows scale‐invariance, or self‐similarity, in the data. The slope of this line is called the scaling exponent ⟨. This scaling exponent ranges from 0.5 (randomness: no correlations present) to 1.5 (regular: no responsiveness). A value of 1 is associated with a ‘healthy’ system that is not too random (0.5), i.e., it reacts to every external influence, and also not too rigid (1.5), i.e., does not react at all to any external influences. More detailed descriptions of this method can be found here (Gu et al., 2015; Peng et al., 1994).

#### 2.2.6 Pre‐sleep rumination

The Rumination Response Scale (RRS) is a self‐report questionnaire designed to assess the presence and the persistence of rumination in response to negative affect (Treynor et al., 2003). It consists of 22 items on a Likert scale format, ranging from 1 (“almost never”) to 4 (“almost always”), with higher scores indicating higher rumination levels. In our study, RRS was adjusted for night‐time use, aiming to assess the levels of pre‐sleep rumination, and was administered only once at the end of the one‐week assessment. The description prior to the questions stated that all the items refer to the evenings of the past week, before participants went to sleep, as previously conducted by Pillai et al. (2014). Participants' pre‐sleep rumination levels were computed by the total score on the RRS. The night version of the RRS achieved excellent internal consistency in the present sample (Cronbach's a = .94).

#### 2.2.7 Covariates

Alcohol and caffeine use were assessed through the sleep diaries (i.e., “How many alcoholic drinks did you have yesterday?”, “How many caffeine‐containing drinks did you have yesterday?”). The total amount of alcoholic and caffeinated drinks was calculated and the summed values across the week were utilized for the analyses. If any of the above variables significantly differed between chronotypes, they were entered into the analyses as covariates.

### 2.3 Procedure

In a face‐to‐face visit, participants were screened with the M.I.N.I. and MEQ and were checked on inclusion/exclusion criteria. If included, participants came to the lab twice. In the first visit, they were given a watch to wear for seven days and nights and a sleep diary to fill out every morning either on paper or digitally. A week later they returned to the lab to bring back the watch and sleep diaries and to fill in the RRS (and other measures not reported here).

### 2.4 Statistical analyses

The statistical analyses were performed using SPSS version 26. Sociodemographic characteristics were compared between chronotypes, using Chi‐square for categorical variables and analysis of variance (ANOVA) for continuous variables. Two multivariate analyses of variance (MANOVAs) were performed to examine the effect of chronotype on subjective and objective SOL, TST and WASO, respectively. Two multivariate analyses of covariance (MANCOVAs) assessed the aforementioned relationships using covariates. Given that our independent variable (i.e., M‐type and E‐type) had only two levels, post hoc tests were not applicable. However, to ensure a rigorous approach to controlling for Type I error, we conducted pairwise comparisons of estimated marginal means (EMMs) with a Bonferroni adjustment. One‐way ANOVA was conducted to assess if the two chronotypes differed in their levels of pre‐sleep rumination. For the (M)ANOVAs, if the assumption of homogeneity of variance was violated, a Brown‐Forsythe *F* test was reported. Two mediation analyses were performed, using PROCESS Version v3.4 (Hayes, 2017), with chronotype as predictor, pre‐sleep rumination as mediator and subjective and actigraphy‐based SOL as dependent variables. Indirect effects and their bootstrapped confidence intervals were the indicators of significance. Two moderation analyses were conducted also with PROCESS, with chronotype as independent variable, depression status as moderator and subjective and actigraphic SOL as dependent variables. Analyses were run with and without covariates. The DFA analysis was performed on the same actigraphy data that was used for the other analyses. The same groups were identified, and the scaling components were compared between groups using independent sample *t*‐tests. We used the time scales ranging from 3 to 8 h, as this was the daily time range where scale‐invariance could be detected reliably.

## 3 RESULTS

### 3.1 Participant characteristics

Table 1 shows the demographic and clinical characteristics of the sample stratified by chronotype. The two chronotype groups did not significantly differ in their age, gender, working status, marital status, education level, depression history or caffeine consumption. Since homogeneity of variance was not assumed for alcohol use: Levene's *F* (1, 83) = 16.43, *p* < .001, a Brown‐Forsythe *F* test showed that E‐types consumed more alcoholic beverages than M‐types (Table 1).

**TABLE 1:** Participants' characteristics stratified by chronotype.

	E‐types	M‐types	Total	
	*N* = 53	*N* = 35	*N* = 88	
	*N* (%)	*N* (%)	*N* (%)	
	*M* (*SD*)	*M* (*SD*)	*M (SD)*	***p* value**
**Sociodemographic characteristics**				
**Age**	21.52 (3.2)	21.51 (4.4)	21.35 (3.7)	.74
**Gender**				.29
Males	5 (9.4)	6 (17.1)	11 (12.5)	
Females	48 (90.6)	29 (82.9)	77 (87.5)	
**Working status**				.57
Employed	29 (54.7)	17 (48.6)	46 (52.3)	
Unemployed	24 (45.3)	18 (51.4)	42 (47.7)	
**Marital status**				.28
Married	1 (1.9)	3 (8.6)	4 (4.5)	
Living together	9 (17.0)	4 (11.4)	13 (14.8)	
Single	43 (81.1)	28 (80.0)	71 (80.7)	
**Educational level**				.67
Secondary school	2 (3.8)	1 (2.9)	3 (3.4)	
Middle‐level college	1 (1.9)	‐	1 (1.1)	
Higher level college	2 (3.8)	3 (8.6)	5 (5.7)	
University	48 (90.6)	31 (88.6)	79 (89.8)	
**History of depression**				.20
Healthy	34 (64.2)	27 (77.1)	61 (69.3)	
History	19 (35.8)	8 (22.9)	27 (30.7)	
**Alcoholic beverages per week**	6.53 (8.3)	1.74 (3.3)	4.61 (7.1)	**<.001**
*Missing*	*2*	*1*	*3*	
**Caffeinated beverages per week**	8.54 (9.2)	7.97 (9.8)	8.31 (9.4)	.79
*Missing*	*1*	*1*	*2*	

*p* value is the result of chi‐square test or ANOVA (Brown‐Forsythe *F* test for alcohol use), adjusted to .05.

### 3.2 Sleep parameters and pre‐sleep rumination

Two MANOVAs, one on subjective sleep parameters (SOL, TST and WASO) and one on objective ones, revealed no multivariate effects (subjective: Pillai's trace = .06, *F* (3, 82) = 1.78, *p* = .16; objective: Pillai's trace = .07, *F* (3, 77) = 1.99, *p* = .12). However univariate effects were observed for SOL (*F* [1, 84] = 4.31, *p* = .04). Estimated marginal means with Bonferroni adjustment showed a reliable difference: Δ*M* = 6.91, *SE* = 3.33, *p* = .04, 95% CI [.29, 13.52]. Table 2 reports the results of univariate outcomes in (M)ANOVAs. For subjective SOL we also conducted an ANOVA with the Brown‐Forsythe *F* test because the assumption of homogeneity of variance was violated, and the result remained significant (*F*[1, 77.02] = 5.68. *p* = .02). The univariate effects of chronotype on TST and WASO were not significant. The repeated MANCOVAs, using alcohol consumption as a covariate, on subjective and actigraphy‐based SOL, TST and WASO, showed the same pattern (Table S1). The DFA analysis showed that the scaling exponent is 1 for M‐types and 0.94 for E‐types, and this difference is significant (*t* [79] = 2.242, *p* = .03) confirming that M‐types have a more ‘healthy’ daily activity and sleep–wake pattern. Finally, the one‐way ANOVA showed no significant effect of chronotype on pre‐sleep rumination (Table 2).

**TABLE 2:** Subjective and actigraphy‐based sleep parameters and pre‐sleep rumination levels stratified by chronotype.

	E‐types	M‐types				
	*N* = 53	*N* = 35				
	*M* (*SD*)	*M* (*SD*)	***F test***	***Df***	***Error df***	***p value***
***Sleep diary***						
**SOL in minutes**	21.43 (18.1)	14.52 (8.4)	5.68	1	77	**.02**
*Missing*	*1*	*1*				
**TST in hours**	7.48 (0.9)	7.76 (0.7)	2.15	1	84	.15
*Missing*	*1*	*1*				
**WASO in minutes**	9.12 (8.1)	9.40 (9.7)	0.02	1	84	.89
*Missing*	*1*	*1*				
***Actigraphy***						
**SOL in minutes**	21.11 (13.4)	14.11 (11.2)	6.08	1	79	**.02**
*Missing*	*5*	*2*				
**TST in hours**	6.66 (1.3)	6.69 (1.0)	0.01	1	79	.92
*Missing*	*5*	*2*				
**WASO in minutes**	28.80 (19.9)	30.90 (20.6)	0.21	1	79	.65
*Missing*	*5*	*2*				
DFA (⟨)*	0.94 (0.12)	1.00 (0.12)				**.03**
*Missing*	*5*	*2*				
***Pre‐sleep rumination***	32.92 (10.9)	30.56 (7.5)	1.21	1	84	.27
*Missing*	*1*	*1*				

*p* values are the result of univariate outcomes in (M)ANOVAs and Brown‐Forsythe *F* test (for subjective SOL) *DFA is based on *t*‐test.

### 3.3 Mediation analyses

Mediation analyses 1 were run with pre‐sleep rumination as a mediator of the relationship between chronotype and subjective and objective SOL. Indirect effects in both mediation models were non‐significant (Table S2). Controlling for alcohol consumption did not change this result (see Table S3). The only association that was significant was path b’: pre‐sleep rumination was related to increased subjective SOL (*b* = .44, 95% CI [.11, .77], *t* = 2.63, *p* = .01), but this effect was not observed with objective SOL.

### 3.4 Moderation analyses

Moderation analyses 2 were run with a history of depression as a moderator between chronotype and subjective SOL (Model 1) and objective SOL (Model 3). These models were run again with alcohol consumption as a covariate (Model 2 and Model 4).

A significant interaction effect was found in Model 1: *b* = −15.25, 95% CI [−29.66, −.84], *t* = −2.11, *p* = .04. The simple slopes analysis showed that history of depression significantly moderated the relationship between chronotypes and subjective SOL. Specifically, E‐types with depression history reported longer SOL [*M* = 28.60, *SD* = 24.79]: *b* = −17.10, 95% CI [−29.33, −4.86], *t* = −2.78, *p* = .01 than healthy E‐types [*M* = 17.30, *SD* = 11.43] and remitted M‐types [*M* = 11.50, *SD* = 4.54]. In Model 2, with alcohol consumption as a covariate and subjective SOL as dependent variable, the simple slope analysis revealed the same pattern: *b* = −17.47, 95% CI [−29.92, −5.02], *t* = −2.79, *p* = .01. Finally, interaction effects on objective SOL were non‐significant both in Model 3: *b* = 7.43, 95% CI [−5.65, 20.51], *t* = 1.13, *p* = .26 and Model 4: *b* = 8.11, 95% CI [−5.15, 21.37], *t* = 1.22, *p* = .23,

## 4 DISCUSSION

The present study revealed that E‐types have a longer subjective and actigraphic sleep onset latency than M‐types, even after controlling for alcohol consumption. DFA analyses of the actigraphy data showed a ‘healthier’ daily activity and sleep–wake pattern in M‐types. No differences were detected in the other sleep parameters that we measured. Pre‐sleep rumination predicted longer self‐reported SOL but did not mediate the association between eveningness and SOL. History of depression moderated the association between eveningness and subjective SOL. Finally, in line with previous findings, E‐types were found to consume significantly more alcohol (e.g., Van den Berg et al., 2018).

This is the first study that shows that E‐types' longer subjective SOL is confirmed by actigraphy SOL data. Literature to date indicates that E‐types report longer subjective SOL compared to M‐types (Kitamura et al., 2010; Tzischinsky & Shochat, 2011) and morningness has also been found to predict shorter subjective SOL (Soehner et al., 2011); our study confirms these associations using subjective and objective measures. After controlling for alcohol use, eveningness remained significantly associated with longer self‐reported and objectively assessed SOL.

Interestingly, compared to the rest of the recorded sleep parameters, subjective and objective SOL measures vividly align. Likewise, Kearns et al. (2023), using a Bland–Altman approach and multilevel modelling, showed agreement between actigraphic and subjective SOL within a ± 15 minutes window, suggesting that both methods tap into the same sleep construct. Complementary evidence emerges from research on interval timing, proposing that humans, when awake are highly accurate in their temporal judgements in a millisecond‐to‐several minutes range, in compliance with Weber's law (Haigh et al., 2021). However, during sleep, subjective time can significantly deviate from actual time, as a function of sleep architecture, pressure, duration, or circadian phase (for review see Ukraintseva et al., 2021). The latter could potentially explain the alignment of self‐reported and actigraphic sleep onset latency, in comparison to the discrepancy in the other sleep parameters, as the former refers to a pre‐sleep experience of time passing. Yet, due to the naturalistic setting, we cannot exclude that participants may have utilized time‐keeping strategies to estimate times before sleep onset.

In line with past studies, no differences in WASO and TST were detected between chronotypes (Martin et al., 2012). Considering that sleep parameters were assessed via a week‐long multi‐method approach, compared to other studies that used single‐night assessments, the current results are unlikely to reflect a Type‐II error.

Pre‐sleep rumination did not differ between chronotypes and did not mediate the association between eveningness and longer SOL. E‐types have previously been shown to experience more repetitive negative thinking than M‐types (Nota & Coles, 2015) and higher cognitive reactivity, especially rumination (Antypa et al., 2017) that could potentially account for their decreased sleep quality as compared to M‐types (e.g., Bauducco et al., 2020; Tian et al., 2019). However, these previous studies did not measure *pre‐sleep* rumination. The reason for the discrepancy is unclear, but it could be hypothesized that rumination levels are higher during the day in E‐types, compared to M‐types, and not necessarily just before sleeping. Another explanation might be that E‐types experience increased physiological arousal (Roeser et al., 2012) which could be higher during pre‐sleep time. Future studies may investigate these hypotheses further.

Pre‐sleep rumination was associated with longer subjective SOL. Similarly, in previous studies, pre‐sleep rumination was related to longer subjective (Pillai et al., 2014) and actigraphic SOL (Pillai et al., 2014; Zoccola et al., 2009). This is of clinical relevance when treating insomnia, emphasizing the role of the perpetuating cycle between sleep‐related negative thoughts (e.g., daytime fatigue) and emotional arousal (Harvey & Payne, 2002; Tian et al., 2019) in sustaining sleep problems.

Even though chronotypes did not significantly differ in terms of past depression status, eveningness was associated with longer subjective SOL as a function of a history of depression, also after controlling for alcohol consumption. Sleep disturbances may be a residual symptom that amplifies the risk of relapse and recurrence (Li et al., 2012; McGlinchey et al., 2016;Mendlewicz, 2009; Zajecka, 2013). Remitted depressed adolescents report worse sleep quality than healthy controls, regardless of chronotype (Keller et al., 2017). Our findings indicate, however, that E‐types with depression history may be more vulnerable to relapse. Targeting eveningness through behavioural phase‐shift interventions combined with chronotherapeutics may be suitable for mood disorder patients with sleep disturbances (Hasler et al., 2016; Jankowski, 2015). This could facilitate a functional recovery and prevent relapse (Mendlewicz, 2009; Zajecka, 2013).

Depression history did not moderate the association between eveningness and objective SOL though. This null finding could reflect that remitted depressed E‐types are more prone to subjective sleep complaints as they have more negative biases about their sleep compared to healthy E‐types (Kaplan et al., 2012).

The current research has the following strengths. History of MDD was assessed with a validated semi‐structured diagnostic interview. Moreover, we used a twofold assessment of sleep, namely actigraphy along with a week‐long sleep‐diary sampling. Although polysomnography (PSG) has long been considered the “gold standard in sleep research, actigraphy has been found to closely approximate PSG recordings in clinical samples such as depressed insomniacs, exhibiting psychometrically compatible properties (McCall & McCall, 2012). Given the sample size of the current study and our aim to evaluate individuals' sleep quality in a naturalistic setting for seven continuous days and nights, actigraphy was deployed for feasibility purposes. In addition, the analysis of actigraphy for self‐similarity/scale invariance has been previously used as an indication of a healthy daily rhythm in humans (Hu et al., 2009) and we expand this research to our sample of chronotypes.

However, aiming for an ecologically valid protocol, physiological or circadian markers of sleep were not recorded, which is a limitation of the present study. Future research is warranted to further explore the physiological signature of circadian rhythms and sleep latency, as their relationship is previously found to account for at least mild depressive symptoms (e.g. Murray et al., 2017). Furthermore, our participants were mostly females (87.5% of the sample). It has been proposed that males at the mean age of our sample (young adults) present greater eveningness than females (Roenneberg et al., 2007). Thus, due to the small representation of males in our research, the results should be generalized with caution to the male population. Furthermore, the night‐time version of the RRS was administered once at the end of the study and assessed pre‐sleep rumination retrospectively (as measured previously in Pillai et al., 2014). Lastly, no multivariate effects of chronotype on sleep parameters were detected. The report of univariate effects was hypothesis‐driven; thus, replication of the current outcomes is warranted.

## 5 CONCLUSIONS

In the current study, eveningness was significantly associated with longer subjective and actigraphy‐based SOL, proposing that E‐types are more prone to a delayed sleep onset than M‐types. This association was moderated by depression status, showing that remitted depressed E‐types presented longer subjective SOL compared to healthy E‐types and remitted M‐types. Hence, this evidence poses E‐types with depression history at greater risk for depression relapse than M‐types.

## AUTHOR CONTRIBUTIONS

**Efthymia Lamprou:** Data curation (lead); formal analysis (lead); software (equal); writing—original draft (equal); writing—review and editing (equal). **Liia M. M. Kivelä:** Writing—review and editing (equal). **Jos H. T. Rohling:** Formal analysis (equal); writing—review and editing (equal). **Johanna H. Meijer:** Writing—review and editing (equal). **Willem van der Does:** Writing—review and editing (equal). **Niki Antypa:** Conceptualization (lead); data curation (lead); formal analysis (lead); investigation (lead); methodology (lead); project administration (lead); resources (equal); software (equal); supervision (lead); validation (lead); visualization (lead); writing—review and editing (equal).

## CONFLICT OF INTEREST STATEMENT

The authors declare no conflicts of interest. The authors alone are responsible for the content and writing of the paper.

### PEER REVIEW

The peer review history for this article is available at https://www.webofscience.com/api/gateway/wos/peer-review/10.1111/ejn.16551.

## Supporting information


**Table S1.** Subjective and actigraphy‐based sleep parameters, stratified by chronotype categories.
**Table S2.** Summary of Statistical Mediation Model.
**Table S3.** Summary of Statistical Mediation Model, with alcohol consumption as covariate.

## Data Availability

The data that support the findings of this study are openly available in dataverse.nl at https://doi.org/10.34894/WC3G0E [activated after the data is in dataverse.nl, after acceptance of publication].

## Footnotes

^1^
Even though no association between chronotype and pre‐sleep rumination was found, we still run a mediation analyses as previously advised when using the Hayes method of bootstrapped intervals and indirect effects as outcomes (Hayes, 2017).

^2^
Even though the depression status did not differ among chronotypes (Table 1), the moderation analyses were hypothesis‐driven, given that sleep complains have been previously linked with depression relapse (Li et al., 2012).
