# Supplementary material for: Chronotype, sleep quality, depression and pre‐sleep rumination: A diary and actigraphy study
Source: Eur J Neurosci. 2024 Oct 5;60(11):6593–604. doi: 10.1111/ejn.16551 (PMC11612836; doi:10.1111/ejn.16551)
Supplement: Supplementary file 1 — Table S1. Subjective and actigraphy‐based sleep parameters, stratified by chronotype categories. Table S2. Summary of Statistical Mediation Model. Table S3. Summary of Statistical Mediation Model, with alcohol consumption as covariate. [file EJN-60-6593-s001.pdf]

Supplementary Tables

Table S1. Subjective and actigraphy-based sleep parameters, stratified by chronotype categories.

	**E-types**	**M-types**				
	N = 53	*N* = 35				
	M (*SD*)	*M* (*SD*)	***F test***	***df***	***Error df***	***p value***
Sleep Diary						
**SOL in minutes**	21.24 (18.3)	14.52 (8.4)	4.21	1	82	**.04**
Missing	*2*	*1*				
**TST in hours**	7.47 (0.9)	7.76 (0.7)	1.01	1	82	.32
Missing	*2*	*1*				
**WASO in minutes**	9.30 (8.1)	9.40 (9.7)	0.004	1	82	.95
Missing	*2*	*1*				
Actigraphy						
**SOL in minutes**	21.60 (13.5)	14.23 (11.3)	4.01	1	75	**.05**
Missing	*7*	*3*				
**TST in hours**	6.71 (1.2)	6.74 (0.9)	0.03	1	75	.86
Missing	*7*	*3*				
**WASO in minutes**	28.50 (20.2)	31.82 (20.3)	0.55	1	75	.46
Missing	*7*	*3*				

p values result from **MANCOVAs with alcohol use as covariate.**

Table S2. Summary of Statistical Mediation Model

Model	Effect of IV on M(a)		Effect of M on DV(b)		Direct Effect(c) of IV on DV			
	Effect (*SE*)	*p*	Effect (*SE*)	*p*	Effect (*SE*)	*p*	Indirect Effect (*SE*) of IV on DV (a x b)	95% CI (a x b)
1 Subjective SOL (n=84)	-2.52(2.20)	.26	.43(.17)	**.01**	-5.90(3.32)	.08	-1.07(1.39)	-4.72 to .52
3 Objective SOL (n=79)	-2.54(2.30)	.27	-.19(.14)	.20	-7.61(2.91)	**.01**	.47(.70)	-.62 to 2.13

IV: Independent Variable (Chronotype), M: Mediator (pre-sleep rumination), DV: Dependent Variable (subjective /objective SOL)

Numbers in bold indicate significant results (p <.05).

Table S3. Summary of Statistical Mediation Model, with alcohol consumption as covariate.

Model	Effect of IV on M(a)		Effect of M on DV(b)		Direct Effect(c) of IV on DV		Indirect Effect (*SE*) of IV on DV (a x b)	95% CI (a x b)
	Effect (*SE*)	*p*	Effect (*SE*)	*p*	Effect (*SE*)	*p*		
2 Subjective SOL (n=83)	-2.94 (2.35)	.22	.44 (.17)	**.01**	-6.10 (3.56)	.09	-1.29 (1.40)	-4.65 to .67
4 Objective SOL (n=76)	-3.53 (2.53)	.17	-.20 (.15)	.17	-7.15(3.19)	**.03**	.71 (.88)	-.51 to 2.88

IV: Independent Variable (chronotype), M: Mediator (pre-sleep rumination), DV: Dependent Variable (subjective / objective SOL).

Numbers in bold indicate significant results (p <.05).
